# Manifestation of Interactions of Nano-Silica in Silicone Rubber Investigated by Low-Frequency Dielectric Spectroscopy and Mechanical Tests

**DOI:** 10.3390/polym11040717

**Published:** 2019-04-19

**Authors:** Chao Wu, Yanfeng Gao, Xidong Liang, Stanislaw M. Gubanski, Qian Wang, Weining Bao, Shaohua Li

**Affiliations:** 1 Department of Electrical Engineering, State Key Lab of Power System, Tsinghua University, Beijing 100084, China; chaowu@uconn.edu (C.W.); stanislaw.gubanski@chalmers.se (S.M.G.); qwang16@mails.tsinghua.edu.cn (Q.W.); baoweining0525@foxmail.com (W.B.); lsh182@163.com (S.L.); 2 Electrical Insulation Research Center, Institute of Materials Science, University of Connecticut, Storrs, CT 06269, USA; 3 State Grid Jibei Electric Power Co. Ltd. Research Institute, Beijing 100045, China; gaoyfer@126.com; 4 High Voltage Engineering, Chalmers University of Technology, SE–412 96 Göteborg, Sweden

**Keywords:** silicone rubber, silica nanocomposites, interface, dielectric response, mechanical properties

## Abstract

Silicone rubber composites filled with nano-silica are currently widely used as high voltage insulating materials in power transmission and substation systems. We present a systematic study on the dielectric and mechanical performance of silicone rubber filled with surface modified and unmodified fumed nano-silica. The results indicate that the different interfaces between the silicone rubber and the two types of nano-silica introduce changes in their dielectric response when electrically stressed by a sinusoidal excitation in the frequency range of 10^−4^–1 Hz. The responses of pure silicone rubber and the composite filled with modified silica can be characterized by a paralleled combination of Maxwell-Wagner-Sillars interface polarization and DC conduction. In contrast, the silicone rubber composite with the unmodified nano-silica exhibits a quasi-DC (Q-DC) transport process. The mechanical properties of the composites (represented by their stress-strain characteristics) reveal an improvement in the mechanical strength with increasing filler content. Moreover, the strain level of the composite with a modified filler is improved.

## 1. Introduction

Silicone rubber composites are nowadays widely used as insulation material in power system components [1,2]. Inorganic fillers are added to polymers to improve the service performance under various operating conditions. One of the often used fillers in silicone based composites is nanometer-sized fumed silica, the addition of which helps to improve their mechanical and electrical properties. It has also been recently pointed out that the performance of a nanocomposite may deteriorate faster under long term exposure to electrical DC stress, as exemplified for nano-silica filled and unfilled crosslinked polyethylene (XLPE) [3], likely due to the changes introduced by the filler to the dynamics of charge transport in the material. A well-known phenomenon in the preparation of nanocomposite materials is the difficulty in uniformly dispersing the filler in the polymer base bulk. Nano-filler particles often tend to agglomerate, ultimately failing to exhibit the expected properties. Moreover, different physical and chemical properties of the inorganic filler and polymer matrix lead to the incompatibility in the interface. In this case the recent developments in high voltage direct current (HVDC) technology have led to new and more stringent requirements on the performance of insulating materials. Great efforts have thus been undertaken in surface modification of nano-filler particles to improve their dispersibility and distributivity, concurrently affecting the compatibility at particle–polymer interface, passivating surface defects and impeding moisture absorption of the fillers [4,5,6,7,8]. In this context, it is desirable to better understand the impact of nano-filler surface modification on the electrical transport as well as mechanical properties of composite materials for electric power applications, especially in silicone rubber-based composites. We therefore use dielectric response measurements, a powerful non-destructive tool for investigating polymer dynamics and polymer-filler interactions for elucidation of the involved charge transport behavior [4,9,10,11,12,13,14,15,16,17,18].

HVDC power systems are characterized by the existence of various voltage transient phenomena, ranging in their frequency contents from extremely low to high frequencies. They yield temporary changes of the electric field distribution within the insulation systems from the steady resistive state to a mixed capacitive-resistive state or even to purely capacitive. These variations of electric field distribution create, in turn, risk for development of breakdown events and long-term degradation. Low frequency dispersion processes, involving charge hopping in regions with weakly bonded interfaces or with signs of local degradation, have been documented at frequencies lower than 10^−2^ Hz [15,16,17,18,19]. We therefore concentrate our efforts on elucidation of charge dynamics and polarization processes in this frequency region, providing information essential for a proper design of HVDC insulation systems.

The dielectric response characteristics of silicone rubber filled with nanometer-sized silica or alumina tri-hydrate (ATH) have earlier been investigated, focusing on the impact of crosslinking agent [20], silica [21] and aluminum trihydrate (ATH) [17,18,22] contents as well as aging [23]. Our previous work revealed that the quasi-direct current (Q-DC) transport process dominates the dielectric response of ATH interface in the low frequency range [17,18]. Differing from the classical Maxwell-Wagner-Sillars interfacial polarization model [24], Q-DC process is caused by the hopping of weakly bonded charge carriers within localized paths. It can be recognized by analyzing results of dielectric response measurement in terms of the Dissado-Hill many body clusters model [13,14,15,16,17,18], wherein interactions between neighboring entities in a composite are considered. Such analyses are however still missing for silicone-silica based nanocomposites and this contribution is aiming to complete this gap. We therefore report on the effect of surface modification of nano-silica filler in silicone rubber at frequencies as low as 10^−4^ Hz. As a reliable characterization of dielectric responses requires the use of the largest frequency range possible, this is particularly the case when low frequency dispersion processes are present. In addition, we tested the mechanical strength of the composites by measuring their stress–strain characteristics. As the mechanical strength of silicone rubber is mainly influenced by the crosslinking status [25,26,27], the effect of crosslinking density and distribution of crosslinking points on mechanical performance were discussed based on microstructure of the model composites.

## 2. Materials and Methods 

### 2.1. Material Fabrication

Poly-methyl-vinyl silicone rubber (110-2, PMV SiR) was purchased from China Bluestar Chenggrand Co., Ltd. (Chengdu, China). The unmodified and modified fumed nano-silica (CAS 112945-52-5) were acquired from Aladin Industrial Inc., Shanghai, China and the crosslinking agent dicumyl peroxide (DCP, CAS 80-43-3) was purchased from Sigma Aldrich (St. Louis, MI, USA). Three groups of samples were prepared in this study, including pure directly vulcanized silicone rubber and silicone rubbers with 10 to 30 parts of unmodified and modified fumed silica filler per hundred parts of rubber (phr), containing 2 phr of DCP. Thirty phr of nano-silica is a typical content providing the desired tracking and erosion resistance performance as well as mechanical properties to electrical insulation systems. The inorganic fillers and DCP were added to silicone rubber in a double-roll mixer with the nano-silica first and then the DCP. The mixing time of silica was 30 and 5 min for DCP. The composites were then placed in a mold and vulcanized at 443 K under 5 MPa for 10 min for preparing samples with thicknesses of 1 and 2 mm. The 1 mm-thick samples were cut into a disc shape with a diameter of 70 mm for the dielectric response measurement, whereas the 2 mm-thick ones were cut into a dumbbell shape [28] for mechanical characterization. Before each measurement, the thickness of the sample was checked by means of a micrometer at five random points and was found to vary by no more than 2%. Table 1 lists the summary of the sample types, where Un_silica and Mo_silica represent the unmodified and modified silica, respectively.

### 2.2. Parameters of Fumed Nano-Silica

#### 2.2.1. Morphology

**Figure 1:**
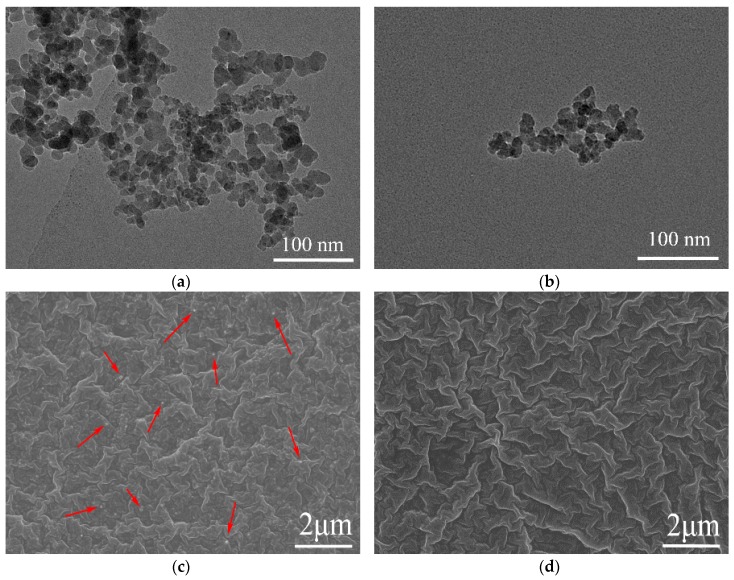
TEM images representing the morphology of unmodified (**a**) and modified (**b**) silica, SEM images representing the morphology of unmodified (**c**) and modified (**d**) silica filled silicone rubber composites.

The specific surface areas of the unmodified and modified silica fillers are 230 and 260 m^2^/g, respectively, characterized using a Brunauer-Emmett-Teller (BET) specific surface area analyzer. The morphology of the silica filled silicone rubber composites was investigated using Transmission Electron Microscopy (TEM) (HT7700) and Scanning Electron Microscopy (SEM) (QUANTA 200 FEG). Figure 1a,b show the TEM images of nano-silica particles, indicating that the individual particle of the silica is around 10~20 nm for both kinds of silica but the particles are often clumped together into larger clusters. The clumped clusters of modified silica were around 100 nm, whereas the agglomerates of unmodified silica were much larger, possibly forming a fractal structure [29]. Although it was proven that such big agglomerates can be dispersed when embedded in the polymer matrix [30], it can be concluded that the unmodified silica can clump together much easier than the modified silica. Figure 1c,d show the SEM images, indicating an improvement in the dispersion of the silica filler particles in the PMV SiR matrix owing to the surface modification. The white points marked in Figure 1c using the red arrows indicate larger agglomerates of unmodified fumed nano-silica particles. In contrast, these are not visible in the SEM images of the modified silicone rubber composite.

#### 2.2.2. FTIR and XPS Analyses

**Figure 2:**
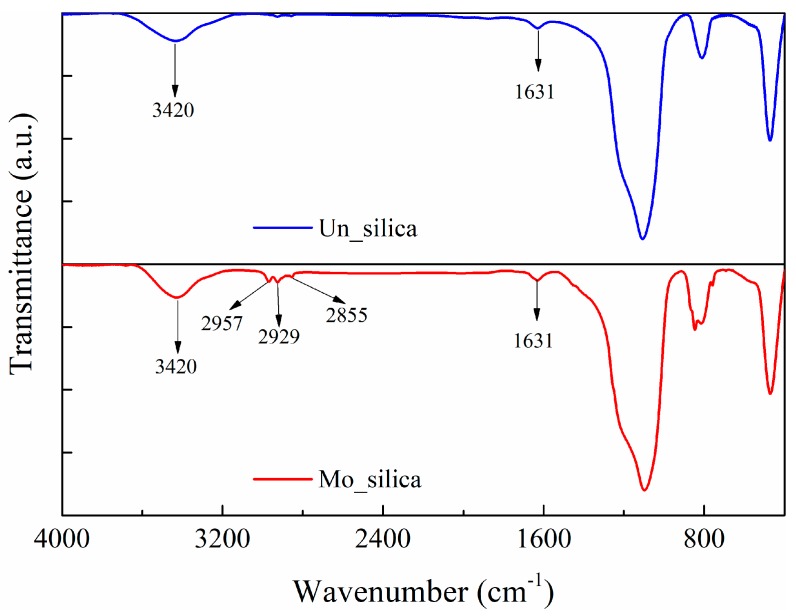
Chemical groups on the surface of unmodified and modified fumed nano-silica fillers investigated by FTIR.

A Fourier Transform Infrared Spectrometer (FTIR) was used to investigate the chemical groups present on the surfaces of the nano-silica particles. The results are illustrated in Figure 2. The three peaks at 2855, 2929, and 2957 cm^−1^ of the transmittance spectrum of the modified nano-silica fillers represent the stretching vibration of C–H groups, indicating the existence of –CH_2_ and –CH_3_ groups on their surface, introduced by the surface modification agent. The wide peak at 3420 cm^−1^ reveals the existence of hydroxyl groups on the surfaces of both types of fillers whereas the small peak at 1631 cm^−1^ represents the bending vibration of the hydroxyl groups. The broad peak in the range of 1000–1200 cm^−1^ and at 810 cm^−1^ is the stretching vibration of Si–O bond.

**Figure 3:**
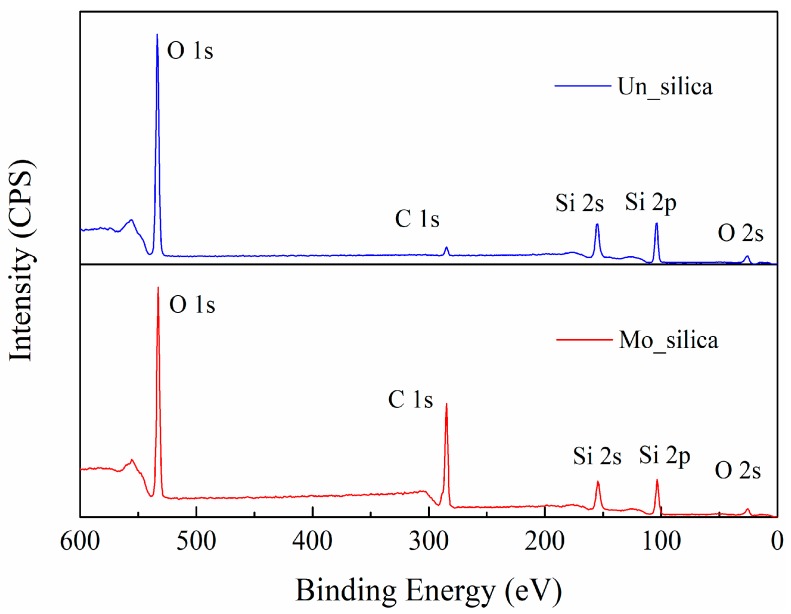
Chemical elements on the surface of unmodified and modified fumed nano-silica fillers obtained by XPS.

Figure 3 shows the chemical elements present on the surface of the nano-silica filler, revealed by X-ray photoelectron spectroscopy (XPS). The pronounced higher intensity of the C(1s) peak of the modified nano-silica fillers confirms the existence of carbon chains introduced by the surface modification. As a commercially available nano-silica was used, FTIR and XPS analyses confirmed that carbon chains dominate on the modified surface of the filler particles and there are no other elements present in addition to silicon and oxygen.

### 2.3. Dielectric Response Measurement

A chamber with the sample and electrode was kept in an oven which allowed the limiting of temperature fluctuation within ±0.5 K throughout the duration of the measurement. The measuring AC voltage level was set at 2.0 Vrms, and the frequency was varied between 10^−4^ to 1 Hz with seven points per decade. The measurement was conducted under three temperatures of 293, 323 and 353 K, which are much higher than the glass transition temperature (*T*_g_) of around 230 K for the silicone rubber composites.

A seven-hour delay before the measurement was set to ensure that the temperature of the sample was the same as the set measurement temperature. At each measurement point, the data was acquired after two voltage cycles. This allows a reliable characterization of the dielectric response and is consistent with the limitations of instrument accuracy. Before the measurements, the samples were cleaned with ethanol and deionized water to remove impurities from their surface. To avoid the presence of water during the measurements, the samples were additionally kept in an oven at 363 K for 10 h before the measurements. In order to ensure that no effect of aging had occurred during the measurement, a preliminary check was made by repeating the measurements at the highest temperature and no differences between the measurements were observed.

### 2.4. Mechanical Strength Measurement

The stress-strain characteristics of the investigated materials were obtained using a tensile testing machine (Zwick Z2.5) under a stretching rate of 500 mm/min. The dumbbell-shaped samples were prepared according to the standard ISO 37: 2005(E) [28], type 1A sample.

## 3. Results

### 3.1. Capacitance and Susceptibility

**Figure 4:**
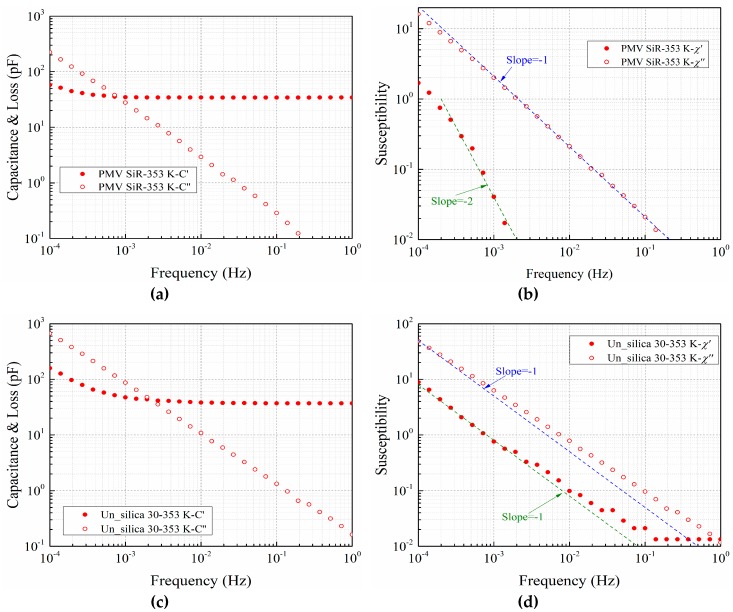

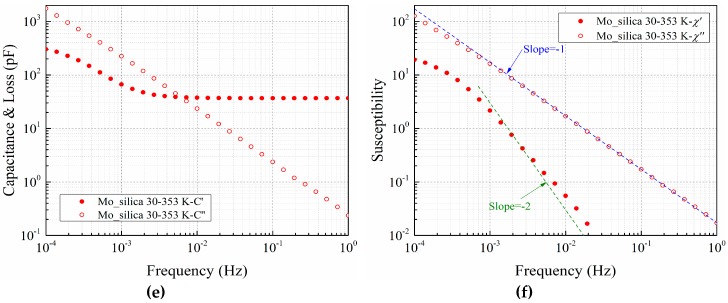
Frequency dependence of complex capacitance and susceptibility of pure silicone rubber (**a**) and (**b**), silicone rubber with unmodified nano-silica (**c**) and (**d**), silicone rubber with modified nano-silica (**e**) and (**f**).

Figure 4 shows the complex capacitance C*(*ω*) and susceptibility χ*(*ω*) of pure silicone rubber (PMV SiR) and silicone rubber with nano-silica in logarithmic coordinates with solid and open dots representing their real and imaginary parts, respectively. χ*(*ω*) was calculated from the measured complex capacitance C*(*ω*) using Equation (1), where *ε*’(*ω*) and *ε*”(*ω*) denote the real and imaginary components of the relative permittivity, respectively. The infinite frequency term *ε*(∞) represents the contribution of the dielectric response in high frequency region beyond the measurement window [31].

(1) $$C^{*}\left(\omega \right)=C^{\prime }\left(\omega \right)-iC^{\prime \prime }\left(\omega \right)=\varepsilon_{0}\frac{A}{d}\left[\varepsilon^{\prime }\left(\omega \right)-i\varepsilon^{\prime \prime }\left(\omega \right)\right]=\varepsilon_{0}\frac{A}{d}\left[\chi^{\prime }\left(\omega \right)-i\chi^{\prime \prime }\left(\omega \right)+\varepsilon \left(\infty \right)\right]\;=\varepsilon_{0}\frac{A}{d}\left[\chi^{*}\left(\omega \right)+\varepsilon \left(\infty \right)\right]$$

The figures show that the results for pure silicone rubber and silicone rubber with surface modified nano-silica are similar among the three types of samples. In the double logarithm coordinate, trend lines with slopes of −1 and −2 are marked in the χ*(*ω*) graphs. In the $\chi^{\prime \prime }\left(\omega \right)$ curves of pure silicone rubber and silicone rubber filled with modified nano-silica, except for the deviation at a frequency approaching 10^−4^ Hz, the data points coincide well with the trend line of slope −1. For $\chi^{\prime }\left(\omega \right)$, the variation trend has a slope approaching −2. In contrast, for the silicone rubber filled with unmodified nano-silica, the shape of χ*(ω) is quite different. The slope of $\chi^{\prime \prime }\left(\omega \right)$ is greater than −1 and is parallel with the slope of $\chi^{\prime }\left(\omega \right)$, which is a characteristic of the Q-DC process in the Dissado-Hill clusters model. In the MWS models, the frequency dependence of the real part of the susceptibility should remain saturate with the decrease of frequency.

### 3.2. Equivalent Circuit Analysis

More than one type of dielectric relaxation process is included in the silicone rubber composites. The C*(*ω*) correlated to individual dielectric processes can be represented as in parallel or in series connected equivalent circuit components, depending on the morphology and composition of the sample.

**Figure 5:**
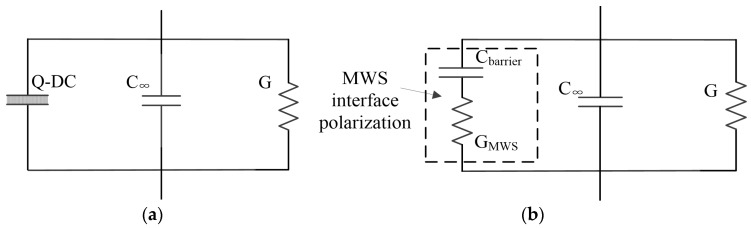
Equivalent circuits of silicone rubber filled with unmodified nano-silica (**a**). Equivalent circuits of pure silicone rubber and silicone rubber filled with modified fumed nano-silica (**b**).

Figure 5a shows the Q-DC conductance process for silicone rubber filled with unmodified nano-silica. In the cases of pure silicone rubber and silicone rubber filled with modified nano-silica, the DC conductance and MWS interface polarization process are included as parallel branches in the equivalent circuit, shown in Figure 5b. 

The functional dependences of $C^{*}\left(\omega \right)$ and $\chi^{*}\left(\omega \right)$ on the MWS polarization can be characterized using Equations (2) and (3).

(2) $$C^{*}\left(\omega \right)=C_{barrier}\frac{1}{1+i\omega \tau }=C_{barrier}\frac{1-i\omega \tau }{1+\omega^{2}\tau^{2}};\left(\tau =\frac{C_{barrier}}{G_{\mathrm{MWS}}}\right)$$

(3) $$\chi^{*}\left(\omega \right)=\chi_{0}\frac{1}{1+i\omega \tau }=\chi_{0}\frac{1-i\omega \tau }{1+\omega^{2}\tau^{2}};\left(\tau =\frac{C_{barrier}}{G_{\mathrm{MWS}}}\right)$$

In the Dissado-Hill clusters Q-DC model, the weak bonded charge pairs can be separated but remain not as absolutely free as in the DC conduction process. Thus, the susceptibility in the Q-DC process follows Equation (4) [19]:

(4) χ*(ω)=χ0Γ(1−p−n)Γ(1−n)Γ(1−p)(1+iωωc)n−1×F12(1−n,1+p;2−n;(1+iωωc)−1)

Here, Γ(a) is the gamma function and _2_*F*_1_(a,b;c;z) is the Gaussian hypergeometric function. $\chi_{0}$ denotes the net concentration of the charge carriers produced by a unit electric field, and $\omega_{c}$ is the characteristic angular frequency. At angular frequencies greater than $\omega_{c}$, the weakly bonded charge pairs move as a coupled dipole over a cluster of sites. At lower frequencies, the charges are so weakly bonded to their counter charges that they can transfer between different clusters. The exponents *p* and *n* (0 < *p*, *n* < 1) characterize the inter-cluster and inner-cluster interactions of the charge carriers, respectively. The index *p* defines the dynamic connection of the charge transport between clusters, whereas the index *n* represents the interactions of charge-pair motion within the clusters. The susceptibility in the Q-DC process can be thus characterized by two power law like responses as follows.

(5) $$\chi^{\prime \prime }(\omega )\propto \chi^{\prime }(\omega )\propto \omega^{n-1},\;\omega >>\omega_{c}$$

(6) $$\chi^{\prime \prime }(\omega )\propto \chi^{\prime }(\omega )\propto \omega^{-p},\;\omega <<\omega_{c}$$

It should be noted that at lower and higher frequency ranges, *x*’ and *x*” become parallel to each other in the log*x*-log*ω* coordinates, and therefore, the Q-DC response is also called a constant phase angle (CPA) response [19].

**Figure 6:**
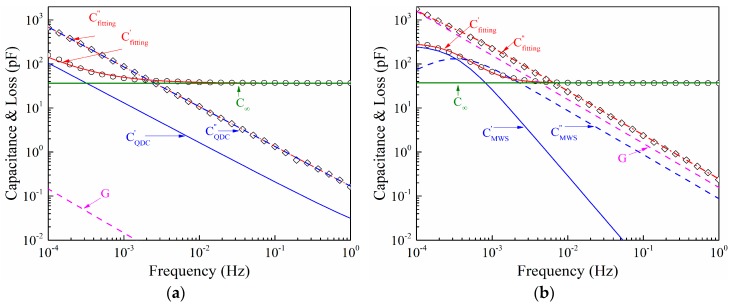
Equivalent circuit fittings for silicone rubber filled with unmodified fumed nano-silica (**a**) and modified fumed nano-silica (**b**) at 353 K.

Figure 6 shows the fittings of the experimental results of the measured complex capacitance with the model circuits for silicone rubber filled with unmodified and modified nano-silica fillers. Table 2 and Table 3 list the employed equivalent circuit parameters representing the resolved terms for complex capacitance of Q-DC, MWS interface polarization, DC conductance, and geometric capacitance. Limited by the data, we can only obtain the parameters related to the lower frequency branch of the Q-DC process. Nevertheless, it clearly reveals the difference between the responses of the silicone rubbers filled with unmodified and modified nano-silica.

### 3.3. Temperature Dependence of Susceptibility

**Figure 7:**
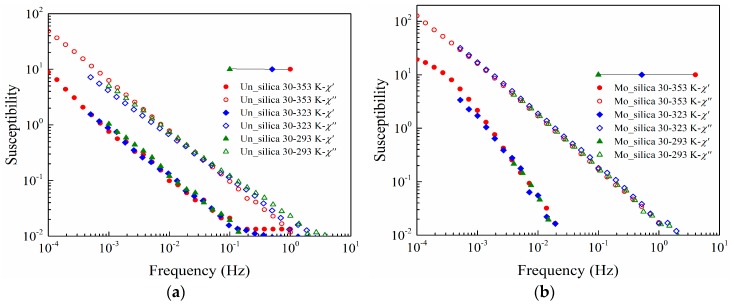
Master curves of silicone rubber composites filled with unmodified (**a**) and modified fumed nano-silica (**b**) at different temperature.

One of the advantages of temperature normalization of dielectric response measurement data is the improved reliability with the increase in the observation frequency range [15,16,17,18,32,33]. To confirm the characteristics discussed above, the dielectric response measurements were repeated at 293 and 323 K. The obtained data at 293 and 323 K are thereafter shifted into the position of data at 353 K, forming so called master curves, as shown in Figure 7. The data at different temperatures for both types of the sample coincide well, indicating that the operating response mechanisms remain the same in the analyzed temperature range.

### 3.4. Mechanical Performance

**Figure 8:**
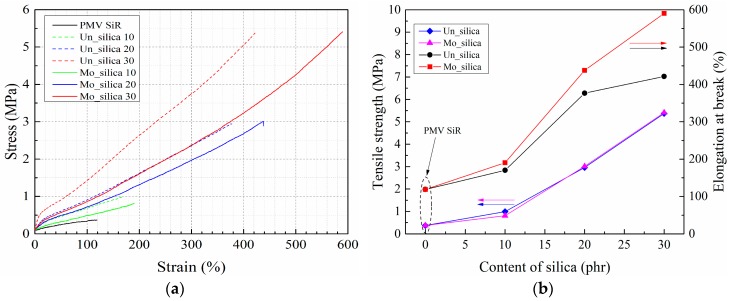
Tensile stress-strain curves (**a**) and mechanical strength (**b**) of silicone rubber composites filled with unmodified and modified nano-silica filler.

Figure 8 shows the stress-strain characteristics of the investigated composites. In Figure 8b, data points, with content of silica being zero, reveal the tensile strength and elongation at the break of the silicone rubber without nano-silica filler. The tensile strength and elongation at the break improve significantly with the addition of fumed nano-silica into the composite system. The comparison of the characteristics of silicone rubbers filled with different contents of unmodified and modified nano-silica shows that the tensile strength remains similar for the unmodified and modified nano-silica composites at the same filler content. However, the elongation at the break becomes significantly greater for the modified fumed nano-silica composites. As the mechanical properties of cross-linked rubbers are mainly influenced by the crosslinking density [27], the bonds formed between the nano-silica filler and the polymer matrix can be treated as physical cross-linking points [25]. In addition, the organic chains on the surface of modified nano-silica can react with the polymer chains of the silicone rubber matrix in the cross-linking reaction. Thus, the increased effective cross-linking density results in higher mechanical strength. The surface properties and dispersion degree of nano-silica can also influence the physical cross-linking effect. A poor dispersion and uneven distribution of the filler particles lead to only localized physical cross-linking regions. The stress will accumulate in these region compared to the polymer matrix regions. In contrast, a good dispersion and distribution of the filler provides a relatively uniform cross-linking, distributing the applied stress in a way that the polymer chains in the network bear similar stress loads [27]. The stress concentration will, on the other hand, result in a break at lower elongations. However, an increasing content of nano-silica increases the possibility of agglomeration, making the observed difference in the elongation at break for samples with 30 parts of nano-silica obvious.

## 4. Discussion

**Figure 9:**
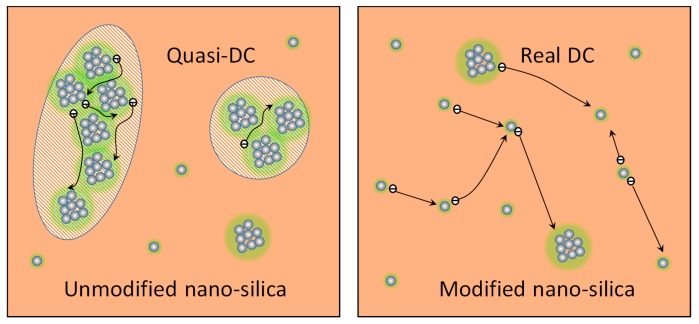
Schematic views of charge interactions in unmodified and modified silica filled silicone rubber composites.

The surface modification by the coupling agent improves the dispersion degree of nano-silica in silicone rubber, which implies greater inter-particle distances. The characteristics of charge transport become similar to those observed in pure silicone rubber, i.e., dominated by the real DC conduction process. In contrast, for the unmodified nano-silica filled rubber, the greater agglomeration of the filler particles and the likely formation of a polymer interphase between them [34], which is presented as the green region around the particles in Figure 9, lead to a Q-DC transport process, wherein the local overlap of the interface provides channels for a localized movement of the charges, as observed in elsewhere [35]. The difference between the behaviors of silicone rubber filled with unmodified and modified nano-silica can be comprehended as follows. The charge transport through the polymer matrix away from the filler particles and the bonded interfacial regions allow a free charge transport process, wherein the motions of single charges do not affect each other. In the agglomerate groups, with overlapping interfacial regions, this motion does not become entirely free because of the interactions with the charges located in the neighboring positions. Such charges can form larger cluster dipoles, the dynamics of which form the Q-DC response.

With regard to the chemical interaction between silicone rubber matrix and nano-silica, the hydroxyl groups present on both types of the filler (broad FTIR peak at 3420 cm^−1^) form hydrogen bonds with oxygen atoms in the siloxane backbone. Moreover, for the modified nano-silica, the interactions between the organic chains in the surface treated nano-silica and the silicone rubber matrix are stronger because of the better compatibility of the organic phases. According to the polymer chain alignment model of [34], a layer of the aligned polymer chains introduced by the surface modification can penetrate into the host polymers and interact with the base polymer chains, forming an interpenetrating network with no free positions for charges hopping. The charges accumulated at the interface are thus transported through the rubber matrix (DC conductance process). In contrast, for the unmodified filler, the weaker interactions between silicone rubber and silica combined with the effect of agglomeration can provide free positions and pathways for charge hopping.

In terms of the mechanical performance, a better dispersion of the physical cross-linking points lead to a more uniform stress distribution in the composite system. On the other hand, the agglomeration of the nano-silica filler particles and the resulting lack of physical crosslinking within the agglomerates contribute toward stress accumulation. In addition, the agglomeration restricts the deformation of the polymer chains [25]. As in the stretching process, the motion of the polymer chains leads to a change in the relative position of the cross-linking points (of both the chemical and the physical ones); it is more difficult to introduce this change in the agglomeration clusters [30]. Moreover, the packed polymer chains in the clusters of crosslinking points yield a steeper slope of the stress-strain curve [27]. We thus conclude that the higher Young’s modulus of the unmodified nano-silica filled silicone rubber composite is a result of the stress accumulation and the reduction in the deformation possibility, but not of the increased cross-linking density.

## 5. Conclusions

The results of dielectric response measurements conducted at low frequency range from 10^−4^ to 1 Hz clearly show how the surface modification of nano-silica filler influences the electric transport mechanism in silicone rubber composites. The charge transport process in modified fumed nano-silica filled silicone rubber composite became similar to that in pure silicone rubber matrix and it followed the DC conduction behavior. In contrast, the composite filled with unmodified nano-silica revealed the operation of the quasi-DC process, in which the frequency dependence of the dielectric susceptibility follows the power law dependence. The agglomeration and thus the overlapping of the interface regions of the neighboring particles in the clusters form local hopping centers, thereby allowing local charge interactions that manifest in the form of a Q-DC process, as proposed in the Dissado-Hill clusters model.

The stress-strain characteristics of the studied silicone rubber composites show a significant improvement in their mechanical strength owing to the formation of additional physical cross-linking points. The improved filler particle dispersion in the surface modified nano-silica composite led to a uniform stress distribution during stretching, yielding a greater elongation at break. However, the tensile strength remained similar for both types of the composites at the same filler content, indicating that the adhesive strength between the polymer chains and the nano-silica particles was not strongly affected. As for the unmodified filler, the agglomeration restricted the deformation of the polymer chains and increased the difficulty in changing the relative position of the filler particles, thereby increasing the Young’s modulus of the composite.

## Figures and Tables

**Table 1:** Filler content and labeling of samples used in tests.

Composition (parts)	PMV SiR	Un_silica x	Mo_silica *x*
PMV SiR	100	100	100
SiO_2_	0	*x* (*x* = 10/20/30)	*x* (*x* = 10/20/30)
DCP	2	2	2

**Table 2:** Parameters of electric circuit elements for silicone rubber filled with unmodified nano-silica.

Circuit Element		Parameters
**Q-DC** **process**	*χ*(0)	8.58 × 10^−4^
	*ω*_c_ (rad/s)	>50 × 2π
	p	0.9
	n	-
G (pS)		9.15 × 10^−5^
C_∞_ (pF)		36.79

**Table 3:** Parameters of electric circuit elements for silicone rubber filled with modified nano-silica.

Circuit Element		Parameters
MWS polarization	C_barrier_ (pF)	265.3
	G_MWS_ (pS)	0.55
	*χ* (0)	19.2
	*ω*_mws_ (rad/s)	1.3 × 10^−2^
G_dc_ (pS)		0.986
C_∞_ (pF)		37.2
